# Strength prediction model of fractured dolomite and analysis of mechanical properties based on PFC3D

**DOI:** 10.1038/s41598-023-40254-x

**Published:** 2023-08-17

**Authors:** Yi Chen, Junying Rao, Changjie Zhao, Yanghao Xue, Chang Liu, Quan Yin

**Affiliations:** 1 School of Civil Engineering, Guizhou University, Guiyang, 550025 Guizhou China; 2 Research Center of Space Structures, Guizhou University, Guiyang, 550025 Guizhou China; 3 Guizhou Provincial Key Laboratory of Rock and Soil Mechanics and Engineering Safety, Guiyang, 550025 Guizhou China; 4 School of Civil Engineering, Hunan City University, Yiyang, 413000 Hunan China

**Keywords:** Civil engineering, Petrology, Mathematics and computing

## Abstract

To investigate the mechanical properties of fractured dolomite, this study analyzed the fracture characteristics (dip angle, length, position, quantity) using the Pearson coefficient and MIC coefficient. Subsequently, the data pertaining to fracture characteristics is preprocessed using a third-degree polynomial, and a three-classification strategy is implemented to improve the logistic regression algorithm to establish the strength prediction model of fractured dolomite. Furthermore, the significance order of the impact of fracture characteristics on rock strength was determined using the numerical simulation software PFC3D, and the dip angle effect was explained from the perspective of internal fracture propagation within the rock. The results show that: (1) When the regularization coefficient *λ* = 10,000, the algorithm has the highest prediction accuracy and the strongest model generalization ability. (2) The numerical simulation analysis software PFC3D can accurately invert rock failure process and characteristics, and the order of influence of fracture characteristics on rock strength is dip angle > length > position.

## Introduction

The construction of underground engineering projects, such as tunnels in dolomite strata, is progressing rapidly. Acquiring a comprehensive understanding of the mechanical properties of dolomite is essential for ensuring the safety of these underground constructions. Presently, rock mechanics research based on the principles of continuum mechanics is advancing towards maturity. However, accurately predicting the process of fracture initiation and propagation in advance remains a challenging task. Additionally, quantifying the combined characteristics of fractures poses significant difficulties, resulting in substantial discrepancies between calculated results and the actual mechanical state of rock masses encountered in practical underground engineering applications. The presence of internal fractures within rock masses stands as one of the primary causes contributing to the degradation of rock strength^1^. Therefore, conducting a detailed investigation into the mechanical behaviour characteristics and fracture diffusion mechanism of fractured rock under load, while exploring the correlation between fracture characteristics and rock strength, holds utmost importance.

At present, a large number of studies on fractured rock have been carried out at home and abroad, mainly including strength analysis strength prediction model and fracture diffusion mechanism^2–8^. In the analytical solution based on rock strength theory, Xinxi et al. improved the Drucker-Prager criterion based on the influence of dry–wet cycles and fracture dip on shale strength^9^. Liu et al. proposed a new strength criterion, namely the minimum potential energy release rate criterion, which can describe the fracture behaviour of materials more accurately^10^. Jiang, M et al. proposed a new strength criterion by analyzing the DEM simulation results. This criterion considers the randomness and spatial variability of cracks and can be used to evaluate the strength and failure behaviour of random cracks in deep rock^11^. In the fractured rock strength prediction model, most of the machine learning algorithms are the theoretical basis, through the search for data experience to establish a prediction model. Zhongping et al. established the shear strength prediction model of the soil-rock mixture by using the fitting of solid test data and numerical test data^12^. Huimei et al. investigated the evolutionary patterns of crack propagation and observed an exponential growth in crack propagation velocity over time. Subsequently, they proposed a rock mass failure prediction model based on the crack propagation velocity^13^. Li et al. proposed an analysis method based on the fracture network model, which can more accurately predict the shear strength of rock fractures in different opening states^14^. The research results on fracture dip angle are the most abundant in the study of fracture diffusion mechanisms based on fracture characteristics. Wei et al. carried out a dynamic uniaxial compression test on 3D-printed fractured rock samples, and then studied the influence of fracture dip angle on dynamic mechanical properties and energy dissipation law^15^. Wang et al. mainly studied the failure characteristics and mechanisms of granite with different joint and fracture dip angles through experiments and numerical simulations^16^. Zhi-yao et al. used RFPA2D software to study the propagation law of noncoplanar overlapping fractures under different dip angles^17^. Some scholars have also studied the characteristics of multiple fractures. Luo et al. studied the failure process of fractured granite samples with different dip angles, widths and lengths under triaxial loading, and revealed the mechanical properties, failure modes and energy transfer laws of granite under triaxial loading^18^ Ping et al. carried out impact compression tests on 45 sets of intact fractured sandstone specimens with different dip angles, and studied the dynamic mechanical properties and energy consumption of fractured sandstone with different dip angles under impact load^19^.

In summary, the existing strength prediction models for fractured dolomite rock often overlook the influence of fracture characteristic factors, and a comprehensive rock strength prediction model that integrates multiple fracture characteristics has yet to be proposed. Moreover, the theoretical calculations and numerical simulations of fracture propagation mechanisms have predominantly remained confined to a two-dimensional framework, resulting in significant deviations from actual working conditions.

Therefore, we consider it essential to propose an improved dolomite strength prediction model that takes into account fracture characteristics and conducts three-dimensional numerical simulation research. In this study, we plan to collect and process dolomite rock samples directly from the work site. The rubbing method will be utilized to quantify the apparent natural fracture characteristics of the rock, and subsequent uniaxial compression tests will be performed to determine the uniaxial compressive strength of the rock. We will employ the Pearson correlation coefficient and the maximum information coefficient (MIC) to establish the correlation between fracture characteristics and rock strength. Building upon these analyses, we will enhance the logistic regression algorithm to develop a fracture characteristics-strength prediction model. Furthermore, using the PFC3D software, we will conduct numerical experiments to evaluate the significant order of influence of fracture dip angle, position, and length on rock strength. Moreover, these experiments will further elucidate the detrimental effects of fracture dip angle on rock strength, providing valuable insights into the weakening characteristics associated with fracture dip angle.

## Fracture characteristics analysis and strength index

**Figure 1:**
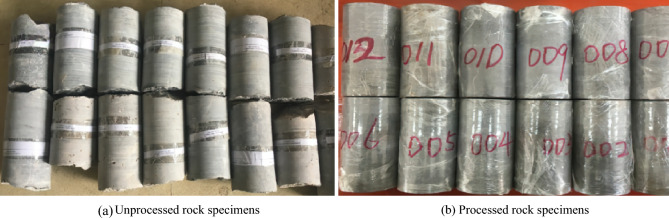
Test rock samples.

In the Guiyang metro tunnel area, field sampling was conducted using a rock core drilling machine with a diameter of 10 cm. The samples were processed to create test rock specimens with a diameter of 50 mm and a height of 100 mm, totalling 21 specimens. Some rock samples are shown in Fig. 1.

**Figure 2:**
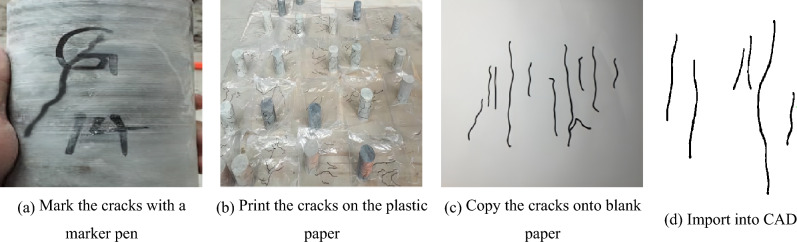
Statistics of microcracks on the rock surface.

**Figure 3:**
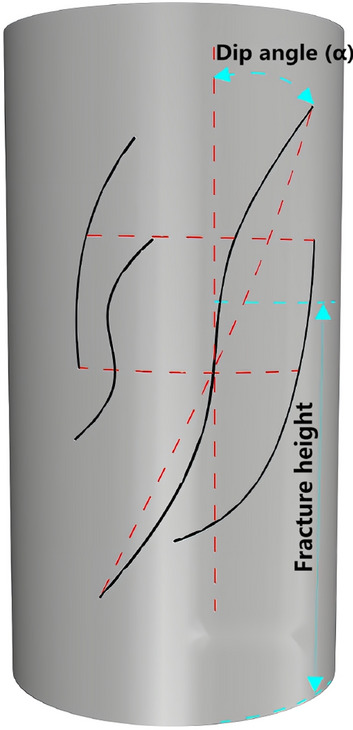
Fracture inclination and height.

**Figure 4:**
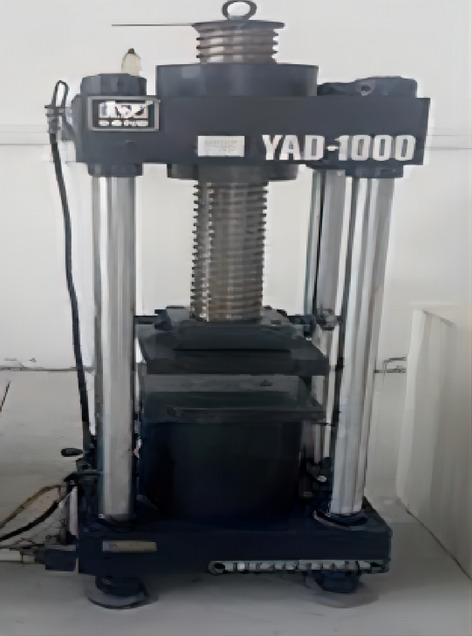
Universal testing machine.

The micro-cracks on the surface of the rock samples were quantified using the rubbing method. Under favourable lighting conditions, the micro-cracks were delineated using a black marker pen. Subsequently, the rock samples were wrapped in transparent plastic paper to capture the cracks. The plastic paper was then stretched to transfer the crack patterns onto white paper, which were later imported into computer-aided design software (CAD) for precise quantitative analysis. The operation steps are shown in Fig. 2a–d. The statistical data of apparent fractures of rock samples are shown in Table 1. The main parameters obtained are fracture number density (total number of fractures/fracture coverage area), fracture length density (total fracture length/fracture coverage area), fracture position and fracture dip angle. Due to the significant disparity in the dispersion of fracture distribution among rock samples, fracture length density and number density are utilized to precisely quantify the fracture length and number, respectively. The fracture location is determined based on the centre height of the fracture concentration area, as illustrated in Fig. 3. The uniaxial compression test was carried out by a universal testing machine (YAD-1000), as shown in Fig. 4, and the strength index is shown in Table 1.

**Table 1:** Apparent fracture characteristics and strength index of rock samples.

Specimen number	Number of fractures (strip)	Total crack length (mm)	Location of the fracture (mm)	Number density (mm mm^−2^)	Length density (strip mm^−2^)	Crack dip angle (°)	Uniaxial compressive strength (MPa)	Strength grade
1	2	79.57	45.5	0.00,012	0.00,466	75	104.21	III
2	3	476.55	56.3	0.00,018	0.02,870	30	62.22	II
3	4	208.92	51.2	0.00,024	0.01,242	0	80.29	II
4	5	281.86	58.2	0.00,030	0.01,679	75	125.17	III
5	6	257.19	61.8	0.00,035	0.01,520	0	117.23	III
6	7	430.69	47.9	0.00,041	0.02,531	15	50.69	I
7	7	437.16	45.1	0.00,041	0.01,590	75	77.76	II
8	7	270.53	51.4	0.00,041	0.02,582	60	90.43	III
9	8	200.25	67.4	0.00,047	0.02,057	90	157.3	III
10	8	350.61	50.8	0.00,047	0.01,188	90	118.61	III
11	9	280.15	48.3	0.00,054	0.01,666	30	63.72	II
12	10	684.98	49.9	0.00,059	0.04,061	15	50.36	I
13	10	635.67	48.5	0.00,058	0.03,718	60	102.35	III
14	11	593.04	63.5	0.00,054	0.03,489	0	98.57	III
15	11	638.17	52.7	0.00,067	0.03,897	15	100.49	III
16	12	333.51	42.7	0.00,071	0.03,719	60	84.56	II
17	12	631.56	53.8	0.00,071	0.01,975	45	63.42	II
18	12	474.87	44.6	0.00,070	0.02,788	90	110.19	III
19	13	649.67	45.8	0.00,076	0.03,804	45	53.95	I
20	13	712.97	52.4	0.00,079	0.04,353	45	61.62	II
21	14	542.21	48.6	0.00,084	0.03,237	30	59	I

## Strength prediction of fractured dolomite

### Correlation analysis of fracture characteristics

Data correlation characteristics can be divided into two categories: linear and nonlinear correlation. For linear correlation analysis, the Pearson correlation coefficient is the most widely employed theory^20,21^. Nonlinear correlation feature analysis involves more intricate theories, but the MIC^22^ based on information theory to extract nonlinear correlation features has wide applicability.

**Figure 5:**
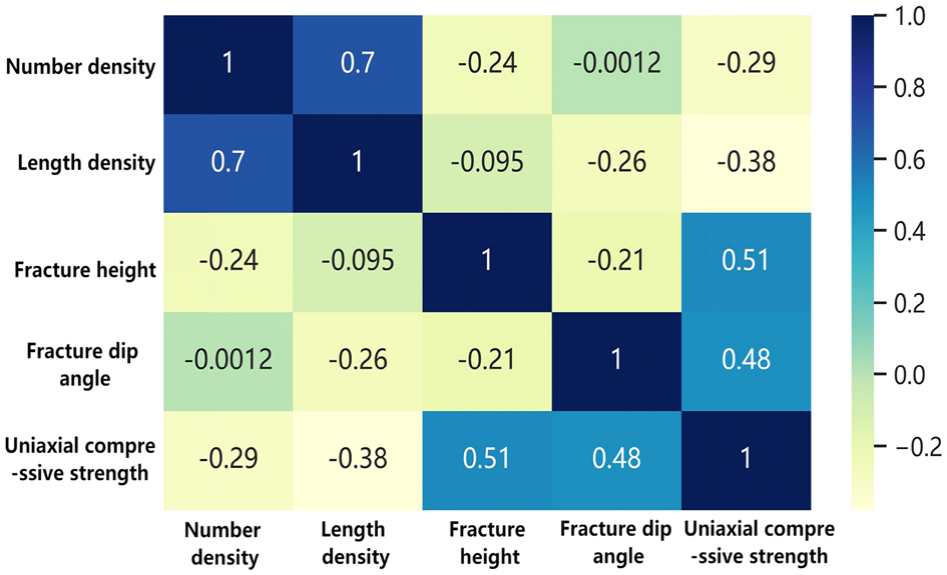
Thermal diagram of Pearson coefficient.

The Pearson correlation coefficient is employed to evaluate the linear correlation between fracture characteristics and the strength index. The theoretical formula is shown in formula (1), and the calculation results are shown in Fig. 5. It can be seen from the calculation results that the correlation between fracture number density and length density is the strongest (0.7), while the correlations among other fracture characteristics are relatively weaker. The order of correlation between fracture characteristics and strength: position > dip angle > length density > number density.

1 $$r = \frac{{\sum\nolimits_{i = 1}^{n} {(X_{i} - \overline{X})} (Y_{i} - \overline{Y})}}{{\sqrt {\sum\nolimits_{i} {(X_{i} - \overline{X})^{2} } \sqrt {\sum\limits_{i} {(Y_{i} - \overline{Y})^{2} } } } }}$$

In formula (1): *X*_*i*_ and *Y*_*i*_ represent the statistical values of two random variables respectively, and $\overline{X }$ and $\overline{Y }$ represent the mean values of the statistical values of two random variables respectively.

The utilization of the Maximum Information Coefficient (MIC) for evaluating the nonlinear correlation between fracture characteristics and strength indexes offers several advantages. MIC encompasses both linear and nonlinear characteristics, with a particular emphasis on capturing and expressing nonlinear features. Moreover, it exhibits robust anti-noise capabilities when handling data with noise. The calculation steps for utilizing MIC are as follows:

1. Suppose fracture characteristic variable *T* = {*t*_*i*_} (*i* = 1, 2, 3, 4) and strength index *S* = {*s*_*j*_} (*j* = 1), *T* represents 4 fracture characteristic variables (number density, length density, dip angle, position), and *S* represents uniaxial compressive strength. The size of mutual information between *S* and *T* is calculated and analyzed by MI. The calculation formula is as follows:

2 $$I_{MI} [T,S] = \sum\limits_{T,S} {{\text{p}}(T,S)} \log_{2} \frac{{{\text{p}}(T,S)}}{{{\text{p}}(T){\text{p}}(S)}}$$

In formula (2), *P*(*T, S*) denotes the joint probability distribution function of T and S, and *P*(*T*) and *P*(*S*) denote the marginal probability distribution function respectively.

2. In the two-digit scatter diagram formed by the data set {*t*_*i*_, *s*_*j*_}, the grid *G* = (*k*, *l*) is drawn to represent the set of grid divisions for the two-dimensional coordinate plane. The horizontal axis is divided into k sub-intervals and the vertical axis is divided into l sub-intervals. All data points must be placed in the divided grid, and the maximum MI of the *G* grid division is calculated according to formula (3).

3 $$I_{MI^{\prime\prime}} [T,S] = \max I_{MI} (T,S,G)$$

3. Normalize the maximum MI value obtained by all meshing schemes, and obtain the maximum value, referred to as MIC, the calculation formula is as follows:

4 $$MIC[T,S] = \mathop {\max }\limits_{|T||S| < B} \frac{{I_{MI^{\prime\prime}} [T,S]}}{{\log_{2} (\min \{ k,l\} )}}$$

In formula (4), *B* = *n*^*0.6*^, n represents the number of samples.

**Figure 6:**
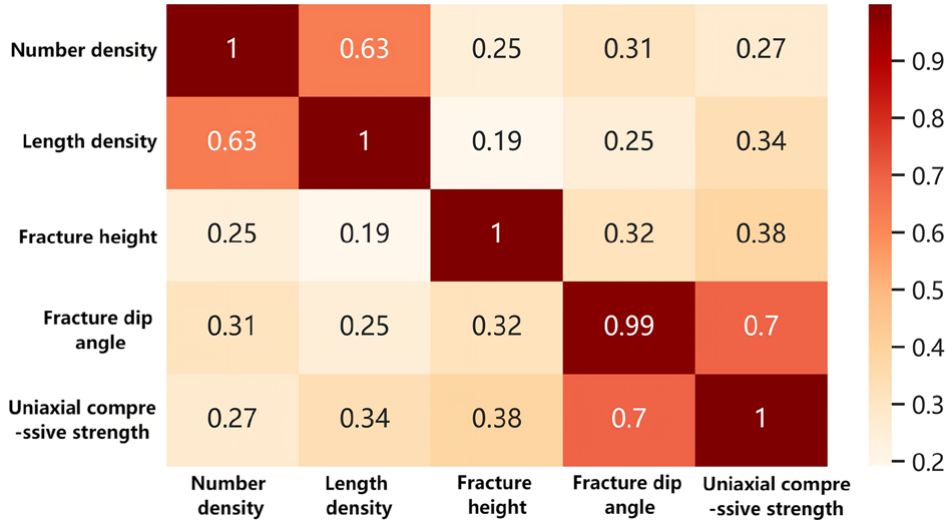
Thermal diagram of MIC coefficient.

**Figure 7:**
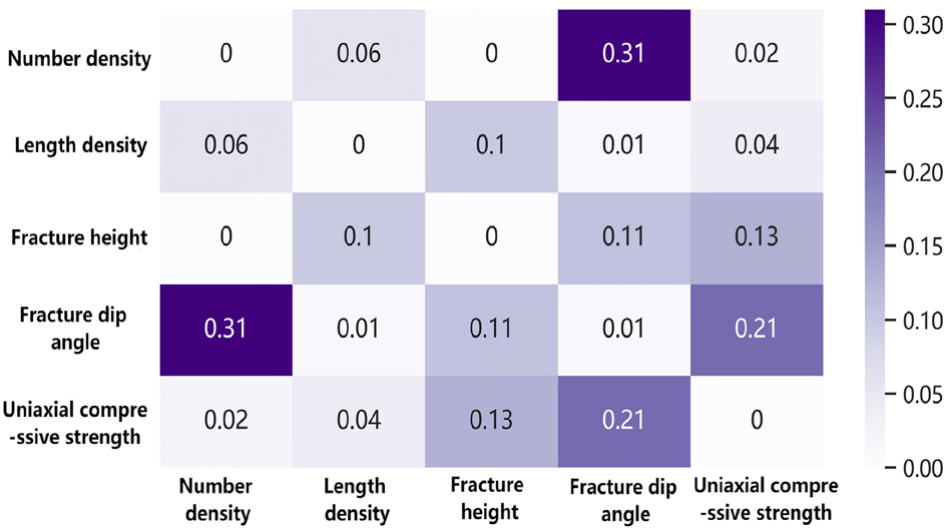
Thermal diagram of the difference between Pearson coefficient and MIC coefficient.

The calculation results based on MIC correlation are shown in Fig. 6. The Mutual Information Coefficient (MIC) has a value range of [0, 1]. A value of 0 indicates complete independence between two variables, while a value of 1 indicates a complete correlation between the variables. According to Reshef's completed research results^23^: The closer the absolute difference between the MIC coefficient and the Pearson coefficient is to zero, the stronger the linear correlation between the variables. Conversely, a larger difference indicates a stronger non-linear correlation between the variables. The calculation results are shown in Fig. 7. The findings indicate the presence of nonlinear correlations between fracture characteristics and rock strength, with the order of significance as follows: dip angle > position > length density > number density. Additionally, there exists a certain level of nonlinear correlation between fracture dip angle and both number density and position. Moreover, a certain degree of nonlinear correlation is observed between length density and position.

The above results indicate the presence of nonlinear characteristics in the relationship between fracture characteristics and rock strength, and there are also correlation characteristics between fracture characteristics. Therefore, employing a linear model for predicting rock strength may result in significant errors.

### Improved logistic regression algorithm

Based on the research findings of the aforementioned fracture characteristics correlations, it is not advisable to use linear fitting to predict rock strength values. Therefore, a fracture characteristics-strength prediction model is established based on the logistic regression classification algorithm^24,25^. Since the logistic regression algorithm is a classification algorithm, it requires categorization of the compressive strength of the rock. According to the *“Specifications for Design Highway Tunnels”* (*JTG D70-2018*), the suggested classification strength threshold for hard rock is 60MPa, and the uniaxial compressive strength of typical dolomite is 90 MPa. Therefore, the strength of dolomite is divided into three grades: Grade I (< 60 MPa), Grade II (60–90 MPa), and Grade III (> 90 MPa), as presented in Table 1.

Logistic regression algorithm mainly through the sigmoid function to deal with data sets into a binary classification problem. The sigmoid function is shown in formula (5).

5 $$\left\{ \begin{gathered} S(x) = \frac{1}{{1 + e^{ - h(x)} }} \hfill \\ h(x) = w_{i} x_{i} ,(i = 1...n) \hfill \\ \end{gathered} \right.$$

In formula (5), *h* (*x*) represents a multivariate linear function, where n represents the data dimension.

**Figure 8:**
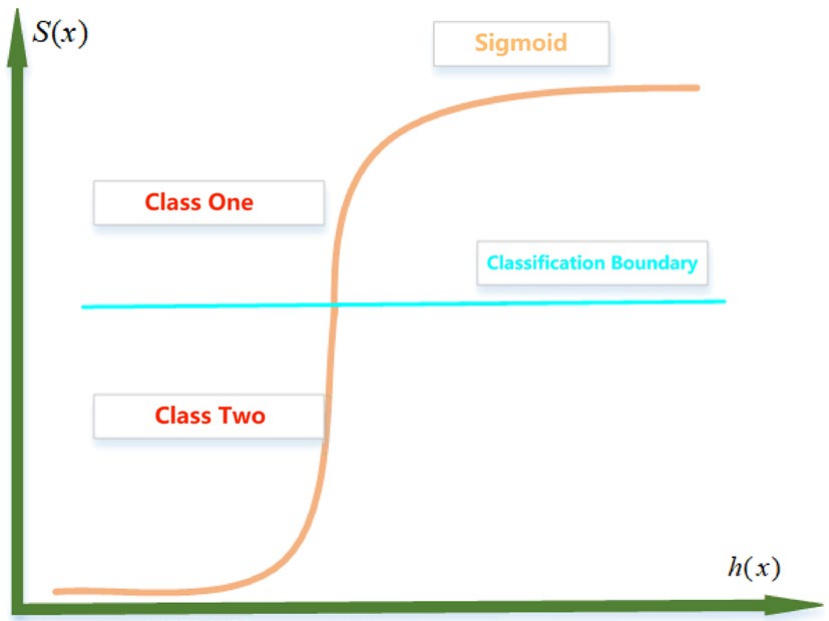
Classification Principle of Logic Regression.

The sigmoid function is employed to transform *h(x)* into a probability expression, where the probability range is [0, 1]. By comparing the probabilities of the data belonging to the first and second categories, we can determine the category to which it belongs. The classification principle is shown in Fig. 8.

**Figure 9:**
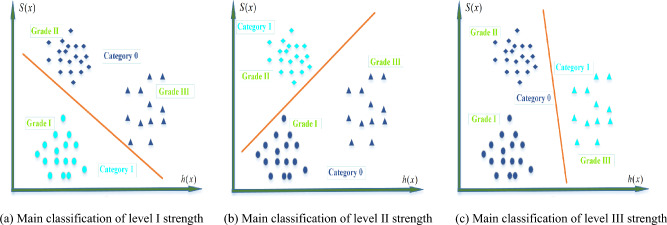
Schematic diagram of three classification models.

Since the strength of dolomite has been categorized into three grades (I, II,III), the logistic regression algorithm, which is typically used for binary classification, needs to be enhanced. To address this, the original data set {*x*, *y*} is copied into three parts, and the two-classification calculation is carried out respectively. The three-category model diagrammatic drawing is shown in Fig. 9. As shown in Fig. 9a, the data is divided into 1 class by I grade rock strength index, and the data of II grade and III grade rock strength index is divided into 0 class, which constitutes the training data set {*x*_*i*_, *y*_*i*_}, and then the type of prediction data is calculated by the formula (6). Similarly, the data is divided into 1 class by II or III grade rock strength index, and the data of the other two grades of rock strength index is divided into 0 class, which constitutes the training data set {*x*_*j*_, *y*_*j*_} and {*x*_*k*_, *y*_*k*_}, and the prediction data category is calculated by the formula (6) again. Finally, the strength grade of the rock is determined by formula (7).

6 $$\left\{ \begin{gathered} S_{w}^{(I)} (x_{i} ) = P(y = 1|x_{i} ;w)(i = 1...n) \hfill \\ S_{w}^{(II)} (x_{j} ) = P(y = 1|x_{j} ;w)(j = 1...n) \hfill \\ S_{w}^{(III)} (x_{k} ) = P(y = 1|x_{k} ;w)(k = 1...n) \hfill \\ \end{gathered} \right.$$

7 $$S_{w} (x) = \max (S_{w}^{(I)} (x_{i} ),S_{w}^{(II)} (x_{j} ),S_{w}^{(III)} (x_{k} ))$$

Based on formula (8), the function *h(x)* represents a first-degree polynomial. However, considering the presence of nonlinear characteristics in both fracture characteristics and rock strength, as well as the nonlinear associations among the feature variables, the influence of fracture characteristics on rock strength involves a multifactor coupling effect. Therefore, it is necessary to perform polynomial conversion on fracture characteristics to improve the prediction accuracy of the model. By employing formula (10), the feature variables undergo a second-degree polynomial transformation with cross-terms. Similarly, formula (11) is applied for a third-degree polynomial transformation with cross-terms. This process can be extended to higher-order polynomial transformations, following a similar procedure.

8 $$h({\text{x}}_{l} ) = w_{l} x_{l}$$

9 $$h({\text{v}}m) = w0 + wmxm$$

10 $$h(v(x)) = \left[ \begin{gathered} 1 \hfill \\ \vdots \hfill \\ x_{l} \hfill \\ \end{gathered} \right]\left[ {\begin{array}{*{20}c} {a_{11} } & \cdots & {a_{1(l + 1)} } \\ \vdots & \ddots & \vdots \\ {a_{(l + 1)1} } & \cdots & {a_{(l + 1)(l + 1)} } \\ \end{array} } \right]\left[ \begin{gathered} 1 \hfill \\ \vdots \hfill \\ x_{l} \hfill \\ \end{gathered} \right]^{T}$$

11 $$h^{*} (v(x)) = \left[ \begin{gathered} 1 \hfill \\ \vdots \hfill \\ x_{l} \hfill \\ x_{1}^{2} \hfill \\ \vdots \hfill \\ x_{l}^{2} \hfill \\ \end{gathered} \right]\left[ {\begin{array}{*{20}c} {a_{11} } & \cdots & {a_{1(l + 1)} } & {a_{1(l + 2)} } & \cdots & {a_{1(2l + 1)} } \\ \vdots & \ddots & \vdots & \vdots & \ddots & \vdots \\ {a_{(l + 1)1} } & \cdots & {a_{(l + 1)(l + 1)} } & {a_{(l + 1)(l + 2)} } & \cdots & {a_{(l + 1)(2l + 1)} } \\ {a_{(l + 2)1} } & \cdots & {a_{(l + 2)(l + 1)} } & {a_{(l + 2)(l + 2)} } & \cdots & {a_{(l + 2)(2l + 1)} } \\ \vdots & \ddots & \vdots & \vdots & \ddots & \vdots \\ {a_{(2l + 1)1} } & \cdots & {a_{(2l + 1)(l + 1)} } & {a_{(2l + 1)(l + 1)} } & \cdots & {a_{(2l + 1)(2l + 1)} } \\ \end{array} } \right]\left[ \begin{gathered} 1 \hfill \\ \vdots \hfill \\ x_{l} \hfill \\ x_{1}^{2} \hfill \\ \vdots \hfill \\ x_{l}^{2} \hfill \\ \end{gathered} \right]^{T}$$

For the data set {*V*, *Y*} obtained after polynomial transformation, where *V* represents the new feature vectors and *Y* represents the class labels (0 or 1), the classification probabilities of the prediction data are calculated using formula (12) to solve for the parameter *w*.

12 $$P({\text{y}}|v,w) = (S(v))^{y} (1 - S(v))^{1 - y}$$

Since the class label y is a discrete number of 0 or 1, the L2 norm cannot be used to define the loss function. Since the final output of the logistic regression algorithm is a probability value, the parameter w is solved based on the maximum likelihood principle. The likelihood function is shown in formula (13). To simplify the solution process, logarithmic transformation is performed, as shown in formula (14).

13 $$L(w) = \prod\limits_{l = 1}^{n} {P(y_{l} |v_{l} ,w)} = \prod\limits_{l = 1}^{n} {(S(v_{l} ))^{{y_{l} }} (1 - S(v_{l} ))^{{1 - y_{l} }} }$$

14 $$\ln (L(w)) = \sum\limits_{l = 1}^{n} {(y_{l} \ln S(v_{l} ) + (1 - y_{l} )} (1 - \ln S(v_{l} )))$$

According to the monotonicity of the logarithmic function, solving the maximum value of formula (14) is equivalent to solving the minimum value of formula (15), and the derivative is obtained by formula (16).

15 $$l(w) = - \sum\limits_{l = 1}^{n} {(y_{l} \ln S(v_{l} ) + (1 - y_{l} )} (1 - \ln S(v_{l} )))$$

16 $$\nabla l(w) = \sum\limits_{l = 1}^{n} {(S(v_{l} ) - y_{l} )v_{l} }$$

Due to the high dimensionality of the feature data *V* and the exponential function *S(x)*, the gradient descent strategy is employed to iteratively solve the formula (17). To ensure that the model has sufficient generalization ability, i.e., robustness, an additional regularization term needs to be added to the formula (17).

17 $$w_{{_{t + 1} }} = w_{t} - \alpha \sum\limits_{l = 1}^{n} {(S(v_{l} ) - y_{l} )v_{l} } - \lambda v_{l}$$

In formula (17), *α* is the learning rate, representing the update rate of the parameter w in the solution process, *α* = 0.0001, *λ* is the regularization coefficient.

### Model validation

Due to the dimensional differences in fracture feature data, formula (18) is used to standardize the data in Table 1. Subsequently, the fracture feature data sets are subjected to polynomial conversions of the 2nd, 3rd, and 4th orders, respectively, resulting in the construction of data sets with different dimensions.

18 $${\text{v}}^{*} = \frac{v - \mu }{\sigma }$$

In formula (17): *μ* represents the average value of data set *V*, and σ represents the standard deviation of data set *V*.

Based on the above theoretical algorithm, using Python programming algorithm to improve the logistic regression algorithm. The prediction accuracy of the model is obtained by substituting the data D1~D21 as the training set 1 into the algorithm. When the regularization coefficient *λ* = 10,000, the prediction accuracy of the model reaches the maximum. The calculation results are shown in Table 2. Clearly, the polynomial transformation has improved the predictive accuracy of the model. Particularly, after applying a 3rd-degree polynomial transformation to the fracture characteristics, the predictive accuracy of training set 1 was relatively the highest. This observation suggests that the 3rd-degree polynomial transformation has maximized the exploration of the nonlinear relationship between fracture characteristics and rock strength.

**Table 2:** Prediction results.

Data set	Range of numbering	Accuracy of prediction			
		Primitive character	Polynomial transformation		
			2 times	3 times	4 times
Training set 1	D1~D21	0.86	0.90	1.00	1.00
Training set 2	D1~D17	0.88	1.00	1.00	1.00
testing set	D17~D21	0.50	0.75	1.00	0.75

The data set D1~D21 is utilized as the training set, which carries a high risk of overfitting and necessitates testing the model's generalization ability. Hence, the data D1~D17 is designated as training set 2 for estimating the model parameters, while the data D17~D21 serves as the test set to evaluate the model's prediction accuracy. The results are presented in Table 2. The prediction accuracy of training set 1, training set 2, and the test set without polynomial transformation is the lowest, indicating an underfitting model. By employing 2nd, 3rd, and 4th-degree polynomial transformations, the prediction accuracy of training set 2 reaches 100%, while the test set exhibits prediction accuracies of 75%, 100%, and 75%. This demonstrates that the model based on the 2nd-degree polynomial transformation is underfitting the characteristic variables, whereas the model associated with the 4th-degree polynomial transformation runs the risk of overfitting. Therefore, it can be concluded that the model utilizing the 3rd-degree polynomial transformation has the strongest generalization ability and the highest prediction accuracy.

## Numerical simulation analysis of fractured dolomite

Due to the existence of nonlinear correlations among fracture characteristics and the significant level of data dispersion in Table 1, it is difficult to evaluate the significance order of the influence of fracture characteristic variables on rock strength by using linear index weight. Therefore, therefore, a numerical experiment is conducted to investigate this matter. As the number of cracks increases, the possible combinations of fracture dip angle, length, and position also multiply, leading to increased complexity and difficulty in defining crack characteristics. Consequently, this study specifically focuses on investigating the significant order of influence exerted by fracture dip angle, length, and position on rock strength.

### Numerical simulation analysis model

In this study, the particle flow numerical simulation software PFC3D^26–28^ is employed to conduct numerical experiments investigating fractured dolomite's mechanical behaviour characteristics and fracture evolution mechanism. In the numerical software PFC, particles are considered to be rigid bodies. The particles themselves are not deformable, and the contact between particles occurs in an infinitely small area. The contact points between particles allow small overlaps, and the size of the overlap is linearly related to the contact force. In the process of rock failure, the parallel bond model between particles will gradually disappear and then degenerate into a linear model. When the parallel bond is effective, the force and torque can be transmitted between the particles. After the failure of the parallel bond, only the force can be transmitted between the particles, and the tangential force is provided by the friction force. The sliding conditions of contact or separation between adjacent particles are determined by the Mohr–Coulomb criterion, and the continuous fracture of the force bond between particles can reflect the diffusion mechanism of the crack.

Firstly, the cylindrical dolomite simulation model with a diameter of 50mm and a height of 100mm is created using the built-in basic unit (particle). Subsequently, the model is assigned a mechanical constitutive model. The mechanical response of particle contact is defined using the parallel bond model (PB model). Following the principle of the uniaxial compression test, the contact between the loading plate and the rock is considered as a smooth rigid contact, and thus, the contact model between the rock and the loading boundary is set as a linear model. Moreover, the smooth-joint contact model is selected as the constitutive equation for internal fracture mechanics.

**Figure 10:**
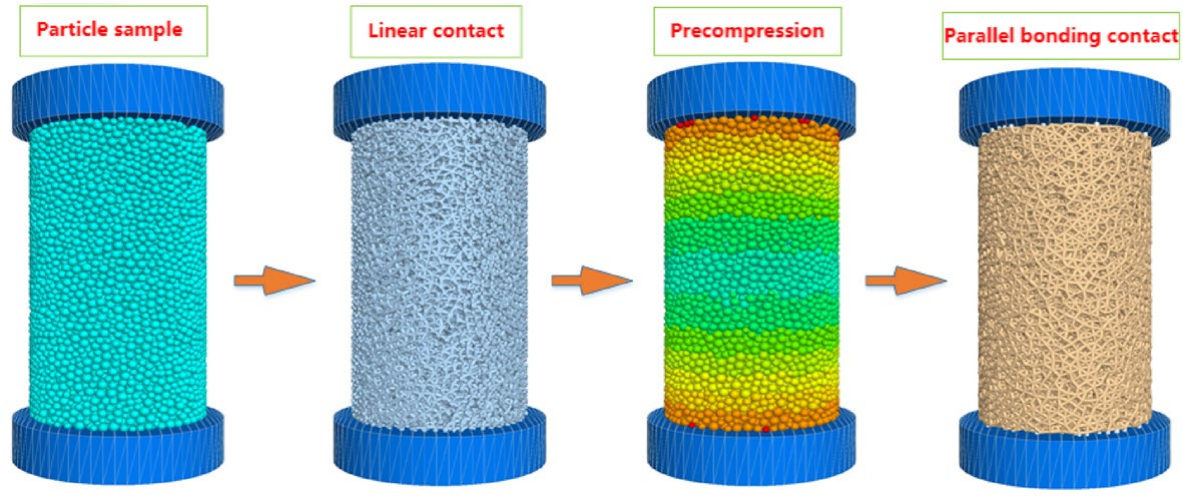
Preloading test of rock numerical model.

To replicate the mechanical state of dolomite under natural geological conditions, it is necessary to subject the simulated rock samples to cyclic preloading and unloading, as illustrated in Fig. 10. The main steps are as follows: (1) Generation of the rock particle model; (2) Definition of internal particle–particle mechanical contact using a linear model; (3) Application of the servo rule to cyclically preload and unload the rock samples; (4) Redefinition of the mechanical response state between internal particles using the parallel bond model.

**Figure 11:**
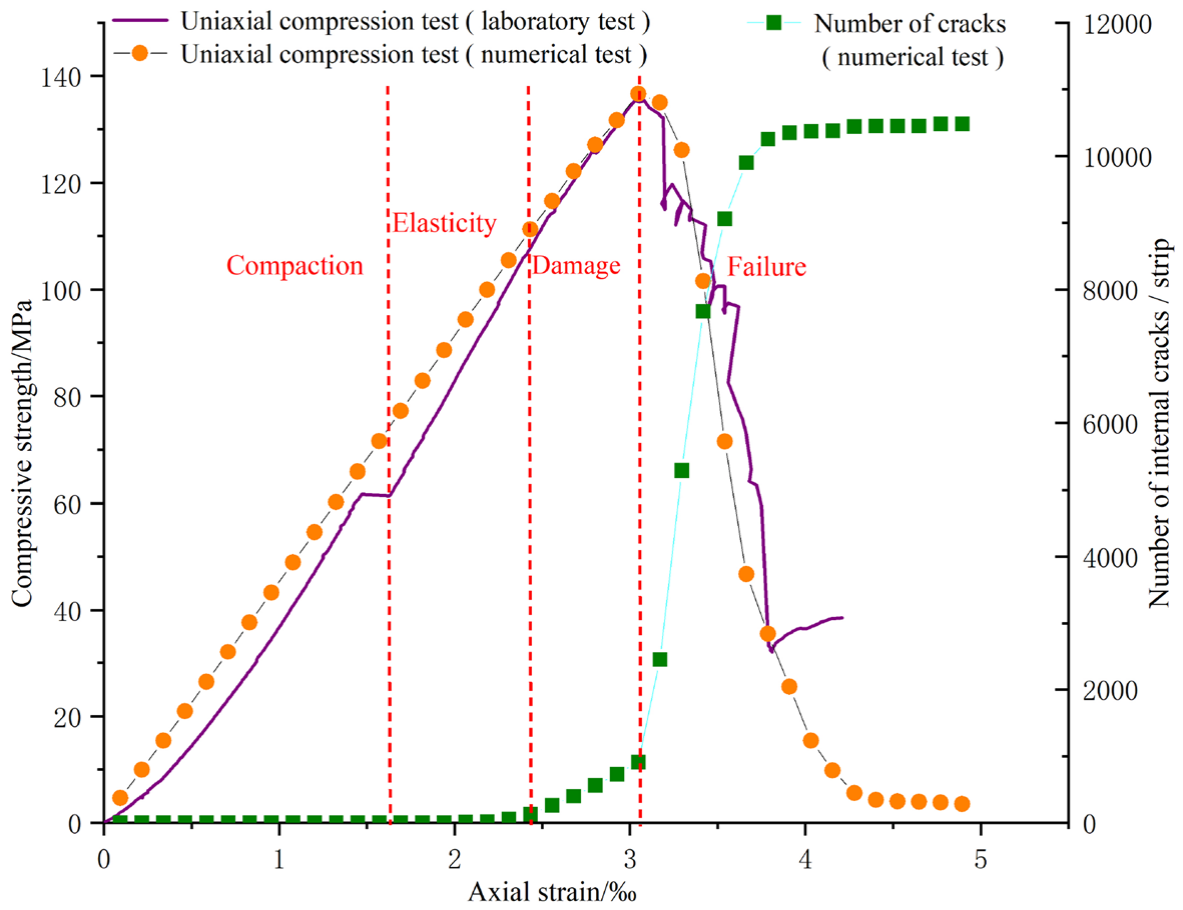
Stress–strain curves for laboratory tests and PFC3D numerical tests.

**Figure 12:**
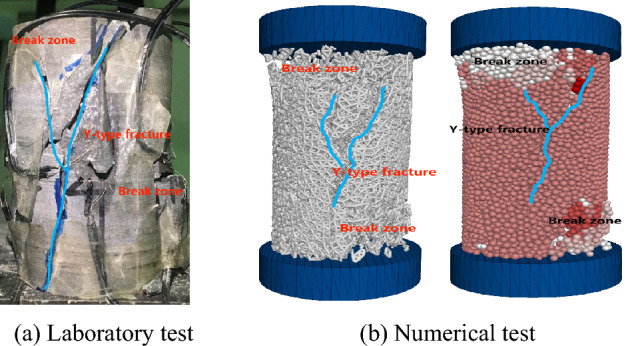
The failure characteristics of rock in laboratory tests and numerical tests.

Due to the numerous physical parameters required for numerical experiments, this paper discusses the similarity between the uniaxial compression test and numerical test of fissureless dolomite by “trial and error method”, so as to invert the experimental parameters required for numerical test, as shown in Fig. 11. The results of both tests show that dolomite has experienced four important stages in the process of uniaxial compression: (1) Compaction stage, during which the stress–strain curve of the physical test exhibits a concave downward trend. As the numerical experiment has carried out the preloading test, the internal contacts are relatively tight; (2) Elastic stage, where the stress–strain curve shows a linear relationship; (3) Damage stage, characterized by the gradual increase of micro-cracks within the rock, which continuously propagate and transform into interconnected fractures; (4) Failure stage, where the compressive strength of the rock reaches its maximum, known as peak strength, followed by a sudden decrease in strength. As shown in Fig. 12, the failure characteristics of the two specimens are essentially similar. Ultimately, effective mechanical parameters can be assigned to the numerical experiment, as presented in Table 3.

**Table 3:** Mechanical parameters of dolomite in laboratory and numerical tests.

Micromechanical parameters of rock					
Density(kg mm^−^^3^)	Elastic modulus(GPa)	Poisson ratio	The angle of internal friction(°)	Cohesion(MPa)	Uniaxial compressive strength(MPa)
2700	42.23	0.34	38.74	20.55	135.50

Smooth-joint contact model parameters					
Normal contact stiffness(GPa)	Shear contact stiffness(GPa)	Friction coefficient	Cohesion(MPa)	Tensile strength(MPa)	Large strain
2	2	0.70	0	0	1

Particle and contact mechanics parameters					
Minimum particle radius(m)	Maximum particle radius(m)	Particle density(kg mm^−3^)	Normal contact stiffness(GPa)	Shear contact stiffness(GPa)	Effective modulus(GPa)
0.0005	0.0008	2700	40	40	40
Friction coefficient	Damping coefficient	Effective cementation modulus(GPa)	Cementing stiffness ratio	Normal bonding strength(MPa)	Shear bond strength(MPa)
0.50	0.50	40	1.50	132	132

### Significance analysis of fracture characteristics

To investigate the significant order of the influence of fracture dip angle, length, and position on rock strength, this study first prefabricates rocks with different fracture characteristics and conducts numerical simulations. The prefabricated fractures are implemented using the built-in smooth-joint contact model. Subsequently, taking the fracture characteristics as the influencing factor and the rock strength as the experimental index, the two-factor analysis was carried out through a comprehensive test to obtain the significant order of the influence of the fracture dip angle, length and position on the rock strength.

**Figure 13:**
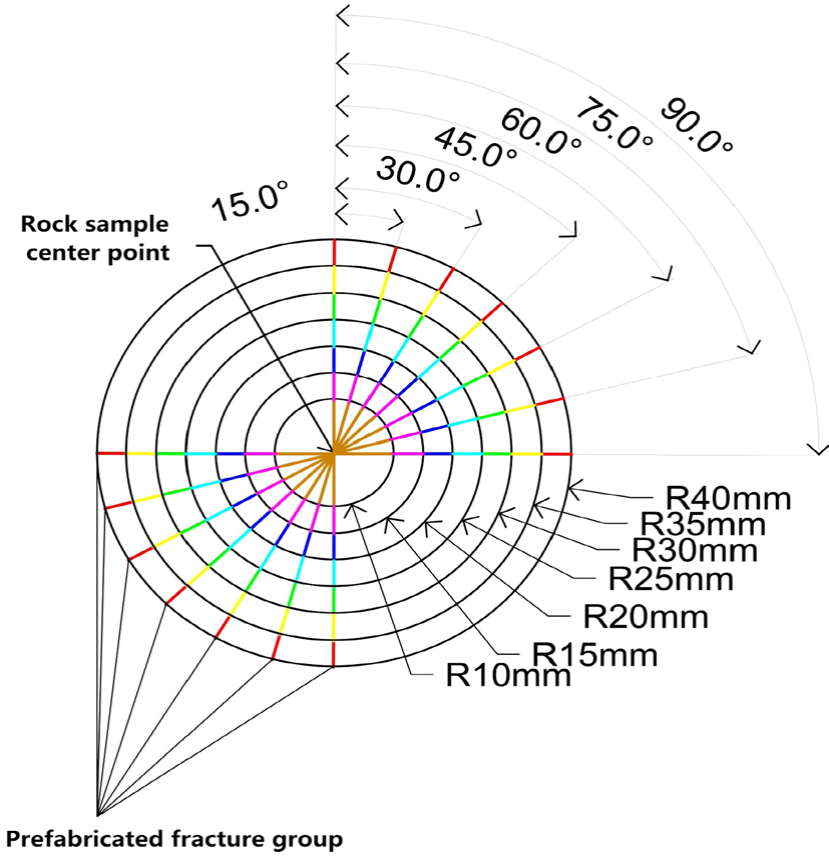
Prefabricated fracture dip angle and length diagram.

**Figure 14:**
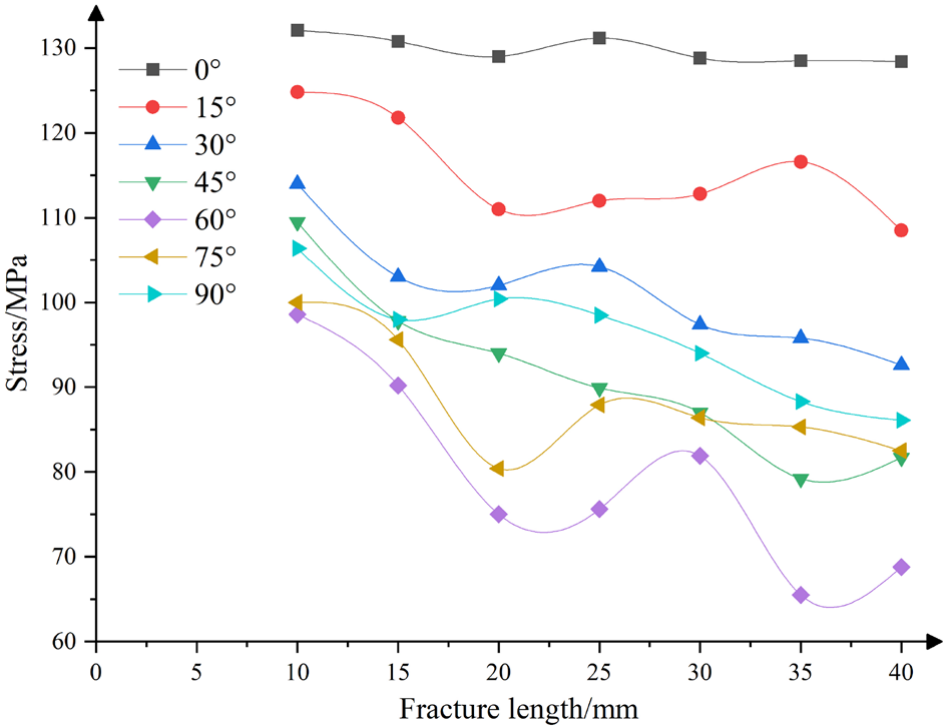
Strength variation characteristics of rock with different fracture angles and lengths.

In order to investigate the significance order of the influence of fracture dip angle and length on rock strength, fractures with various dip angles and lengths were prefabricated, as shown in Fig. 13. The dip angles *α* were selected as 0°, 15°, 30°, 45°, 60°, 75°, and 90°, and each dip angle corresponded to lengths of 10 mm, 15 mm, 20 mm, 25 mm, 30  mm, 35  mm, and 40  mm, resulting in a total of 49 rock samples. The numerical test results are shown in Fig. 14. Subsequently, a comprehensive test analysis of two factors and seven levels is carried out, and the values of dip angle and length correspond to seven levels. The test results show that for a single crack length, the crack inclination angle changes from 0° to 90°, forming 7 test groups. The strength extreme deviation of the test groups is 33.5 MPa, 40.6 MPa, 54 MPa, 55.6 MPa, 46.9 MPa, 63 MPa and 59.6 MPa, respectively, and the average extreme deviation is 50.5 MPa. For a single crack dip angle, the crack length increased from 15 to 40 mm, and the strength extreme deviation of the seven test groups is 3.7 MPa, 16.3 MPa, 21.4 MPa, 30.3 MPa, 33.1 MPa, 19.6 MPa, 20.3 MPa, respectively, and the average extreme deviation is 20.7 MPa. Comparing the average extreme deviation, it can be seen that the influence of fracture dip angle on rock strength is greater than that of fracture length.

**Figure 15:**
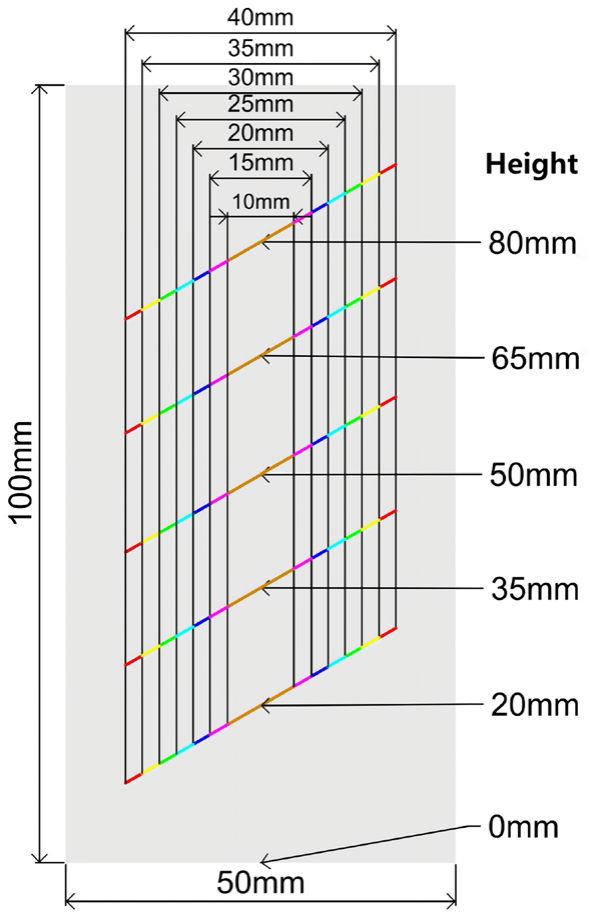
Diagram of length and position of prefabricated cracks.

**Figure 16:**
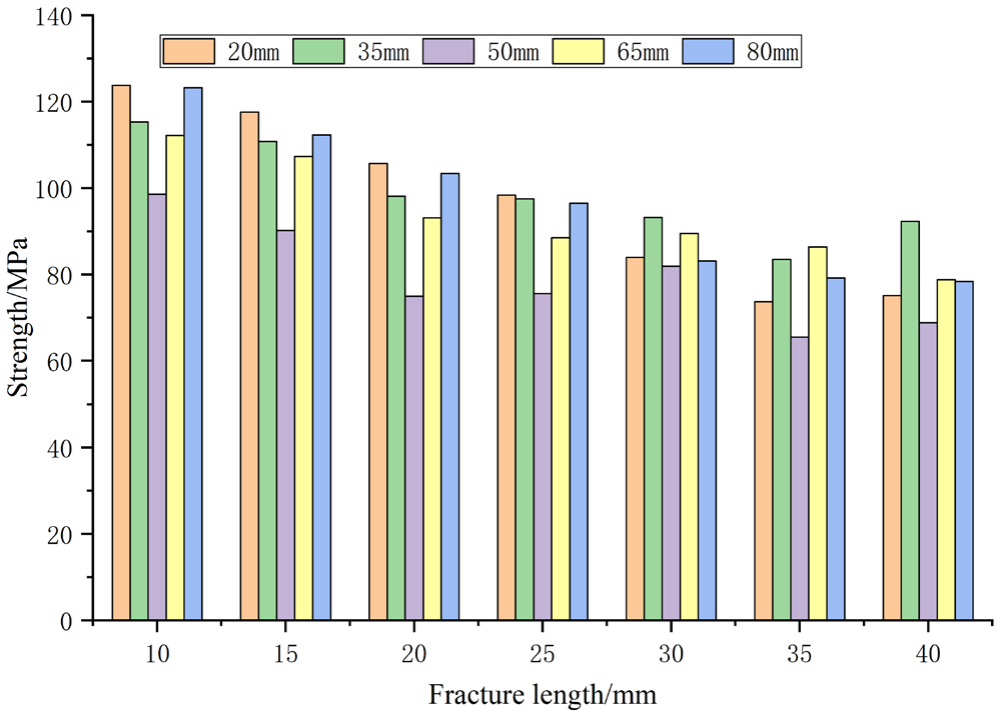
Strength variation characteristics of rock with different fracture lengths and positions.

Similarly, fractures with different lengths and positions were prefabricated, as shown in Fig. 15. The positions of the fractures were set at 20 mm, 35 mm, 50 mm, 65 mm, and 80 mm, and each position corresponded to lengths of 10 mm, 15 mm, 20 mm, 25 mm, 30 mm, 35 mm, and 40 mm. The dip angle of the fractures was set at 60°, resulting in a total of 35 rock samples. The numerical experiment results are presented in the column chart in Fig. 16. When focusing on the fracture position as the main research objective and considering fracture lengths between 10 and 25 mm, the rock strength exhibits a decreasing-then-increasing trend. This suggests that the weakening effect of fractures located in the middle of the rock mass is most significant. In other words, as the distance between the fracture and the loading boundaries increases, the reduction in strength becomes more pronounced. For fracture lengths between 30 and 40 mm, the rock strength does not follow a consistent pattern due to the influence of boundary effects. However, the weakening effect of fractures located in the middle of the rock mass remains most significant.

Furthermore, comprehensive experimental analysis was conducted to determine the significant order of the effects of fracture length and position on rock strength. The results indicate that for each fracture position, with fracture lengths ranging from 10 to 40mm. The strength extreme deviation of the corresponding test groups is 50.1 MPa, 31.8 MPa, 33.1 MPa, 33.4 MPa and 44.9 MPa respectively. The average extreme deviation is 38.7MPa. For each fracture length, seven test groups will be formed. The fracture position of each test group is 20 mm ~ 80 mm. The strength extreme deviation of the corresponding test groups is 25.2 MPa, 27.4 MPa, 30.7 MPa, 22.8 MPa, 11.3 MPa, 20.9 MPa, 23.5 MPa, and the average extreme deviation is 23.1 MPa.

According to the above results, the significant order of the influence of fracture characteristics on rock strength is dip angle > length > position.

### Fracture dip angle effect

**Figure 17:**
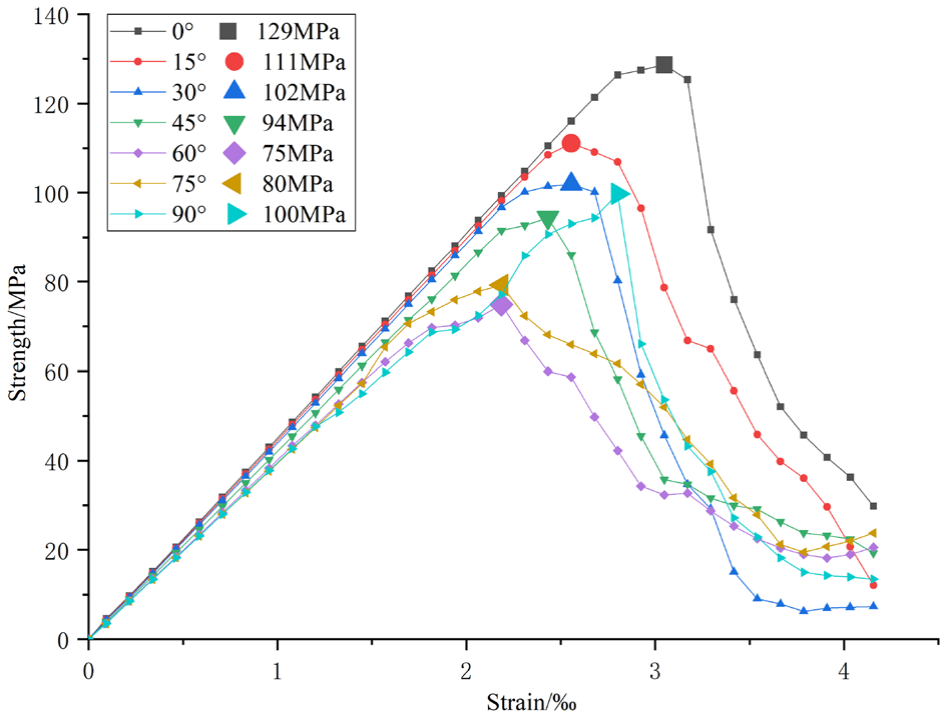
Stress–strain curves of different inclination angles of prefabricated cracks.

Given that the dip angle of the fracture has the most pronounced impact on rock strength, a rock sample with a dip angle *α* ranging from 0° to 90° and a fracture length of 20mm is selected for analysis. The stress–strain curve results are depicted in Fig. 17.

The data in Fig. 17 indicates that when α = 0°, the rock reaches its maximum peak strength (129 MPa). When *α* increases to 60°, the peak strength gradually decreases to 75 MPa, with α values of 15°, 30°, and 45° corresponding to strengths of 111 MPa, 102 MPa, and 94 MPa, respectively. As *α* continues to increase from 75° to 90°, the peak strength rises from 80 to 100 MPa, this indicates that *α* = 60° is the most unfavorable fracture dip angle.

**Figure 18:**
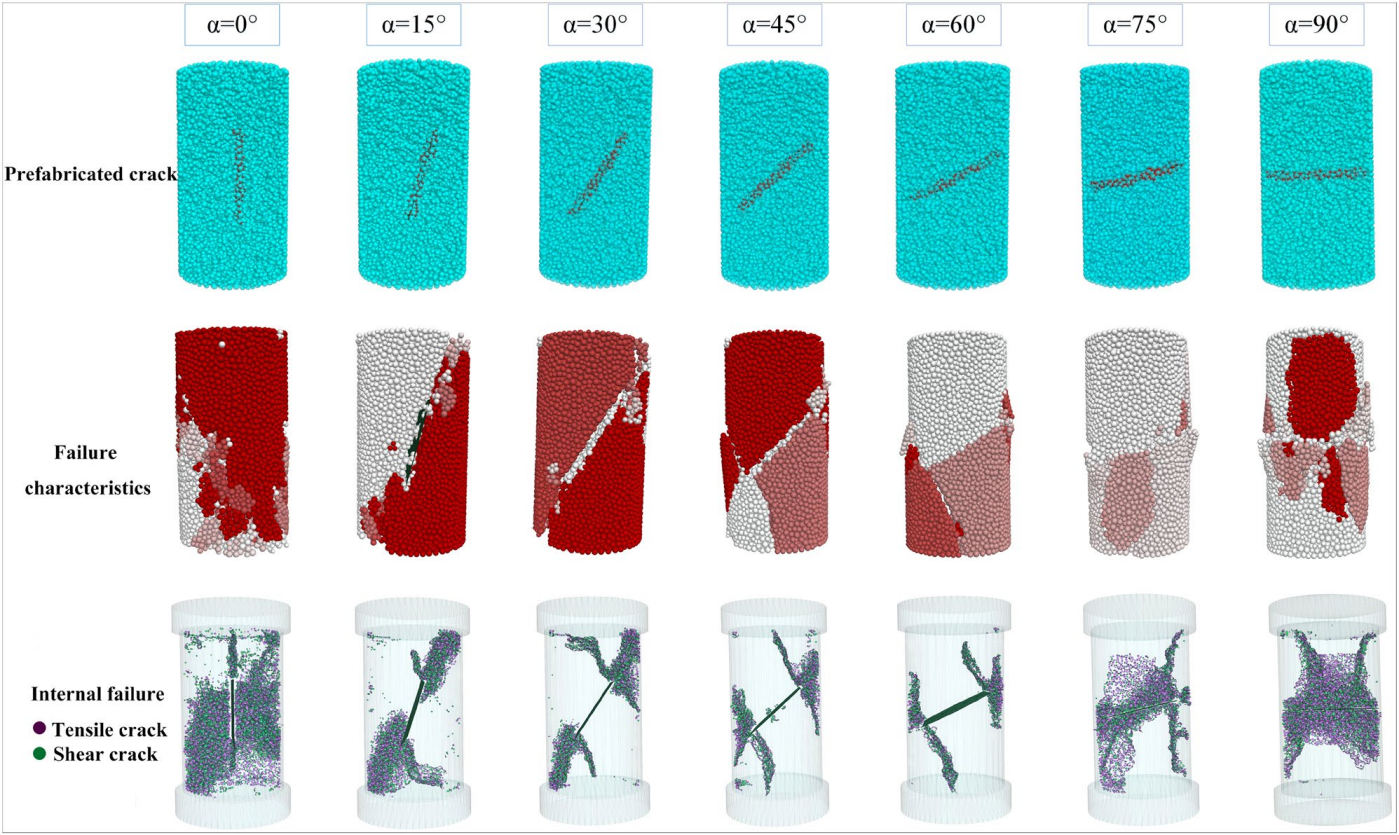
Failure characteristics and fracture development of rock with different dip angles.

The failure characteristics and internal cracks appear in the rock when it reaches its peak strength, as shown in Fig. 18. It can be visually observed that as the rock becomes more fragmented, the block-like fracture characteristics become more pronounced. Moreover, under different fracture dip angle conditions, the number of tensile cracks within the rock is greater than the number of shear cracks, indicating that tensile failure is stronger predominant than shear failure. When *α* = 0°, the rock exhibits the most prominent failure characteristics, with the main fracture zone concentrated at the ends. Based on the information on internal cracks within the rock, it can be inferred that the imaging of the wing-shaped cracks at both ends of the prefabricated fractures is not evident, and the fragmentation of the rock is mainly due to secondary cracks generated by tensile and shear actions. For fracture dip angles ranging from 15° to 60°, from the failure characteristics, it can be seen that the degree of rock fragmentation gradually decreases, and the propagation ability of the wing-shaped cracks at both ends of the prefabricated fracture gradually increases. At the same time, the number of secondary cracks decreases, and the concentrated area migrates from the ends towards the middle section. When *α* = 60°, the characteristics of wing-shaped cracks are most pronounced, with the fewest secondary cracks. For fracture dip angles ranging from 60° to 90°, the wing-shaped crack characteristics gradually weaken, and the secondary cracks increase and concentrate in the middle section of the rock, resulting in severe damage in the middle position.

This can explain the influence mechanism of fracture dip angle on dolomite strength. Under different conditions of prefabricated fracture dip angles, the tensile effect is greater than the shear effect in the process of rock failure. The more the number of secondary cracks produced by tensile and shear failure of rock, the worse the expansion ability of wing cracks, the more small fragments produced by rock fragmentation, but the greater the peak strength of the rock. Conversely, the more obvious the wing fracture characteristics at both ends of the prefabricated fracture, the fewer secondary cracks in the rock, and the fewer small fragments are produced during the rupture process, but the smaller the peak strength of the rock.

### Discussion

In this study, the fracture characteristics were considered as the influencing factors, while the rock strength served as the test index. Through comprehensive test analysis, the significant order of the impact of fracture characteristics on rock strength was determined as follows: dip angle > length > position.

Considering the significant influence of fracture dip angle on rock strength, we selected rock samples with a dip angle ranging from 0° to 90° and a fracture length of 20 mm for further analysis through numerical experiments to investigate the influence mechanism of fracture dip angle on the strength of dolomite. Due to the limitation of paper length, this study did not conduct a more in-depth analysis of the length and position of the fractures. Future research can further investigate the influence mechanism of these two factors on rock strength.

## Conclusion

According to the fracture dolomite strength prediction model and numerical simulation analysis test results, the following conclusions can be drawn:

1. In this paper, the Pearson coefficient and MIC coefficient are used to analyze the correlation of experimental data. It is found that there is a nonlinear correlation between fracture characteristics and rock strength, which indicates that the linear model is not suitable for predicting the strength value of fractured dolomite, and the linear weight is difficult to evaluate the significance order of the influence of fracture characteristic variables on rock strength. Therefore, based on the logistic regression algorithm, this study applies the three-classification model and combines the third-order polynomial conversion strategy to solve the nonlinear characteristic problem of the data, and establishes the fracture dolomite strength prediction model.
2. Numerical experiments are carried out to demonstrate the significance order of the influence of fracture characteristics on rock strength: dip angle > length > position, which is consistent with the conclusion that fracture dip angle has the strongest correlation with rock strength in correlation analysis. At the same time, by comparing and analyzing the uniaxial compression test and numerical test of the entity, it is found that PFC3D can better invert the rock failure process, and can also intuitively reflect the three-dimensional expansion process of internal cracks.
3. For underground engineering projects such as tunnels dominated by dolomite, the strength prediction model of fractured dolomite can be used to estimate the strength of dolomite at the construction site. At the same time, combined with PFC3 D numerical test, more comprehensive and detailed mechanical response data of dolomite are obtained. The research results can further improve the relevant knowledge system of dolomite and provide guidance for the safety of engineering construction.

## Data Availability

The datasets used and/or analyzed during the current study are available from the corresponding author on reasonable request.
